# Emotional Eating in Hispanic Girls and Boys: The Role of Anxiety and Sleep Quality

**DOI:** 10.3390/nu17091588

**Published:** 2025-05-05

**Authors:** Norma Olvera, Tamal J. Roy, Rhonda Scherer, Molly R. Matthews-Ewald, Weihua Fan, Consuelo Arbona

**Affiliations:** 1 Department of Psychological, Health, & Learning Sciences, University of Houston, Houston, TX 77204, USA; tjroy@cougarnet.uh.edu (T.J.R.); rlschere@central.uh.edu (R.S.); wfan@central.uh.edu (W.F.); consueloa@central.uh.edu (C.A.); 2 WhitworthKee Consulting, LLC, Washington, DC 20003, USA; mmatthewsewald@whitworthkee.com

**Keywords:** emotional eating, sleep, anxiety, Hispanic, girls, boys

## Abstract

**Background/Objective:** Emotional eating is a significant health problem associated with increased obesity and mental health among children and adolescents. Investigating emotional eating and its associated factors is critical, as it coincides with key developmental periods during which eating patterns are formed. This study assessed the contribution of anxiety and sleep quality to emotional eating among 232 Hispanic girls (*n* = 124, with a mean age of 10.23 years, *SD* = 1.40) and boys (*n* = 108, with a mean age of 10.36 years, *SD* = 1.57). **Methods:** This study used a correctional research design. Participants completed a series of surveys including demographics, acculturation, McKnight Risk Factor Survey-IV emotional eating subscale, Multidimensional Anxiety Scale for Children, and Pittsburgh Sleep Quality Index. Participants also had their objective body height and weight measured. **Results:** Descriptive analyses showed that most girls (84%) and boys (87%) were born in the United States and were either overweight (*n* = 24, 19% girls; *n* = 18, 17% boys) or with obesity (*n* = 61, 49% girls; *n* = 61, 56% boys). The hierarchical regression analyses revealed that, for girls, poor sleep quality was the sole significant factor associated with EE (*β* = 350, *p* < 0.001), controlling for age and BMI. For boys, poor sleep quality (*β* = 0.302, *p* < 0.01) and anxiety (*β* = 0.247, *p* < 0.05) were significant. **Conclusions:** The study’s findings suggest that emotional eating interventions may need to focus on reducing anxiety levels and improving sleep quality in Hispanic children and early adolescents

## 1. Introduction

Emotional eating (EE), while not a separate clinical eating disorder, is an eating behavior that is characterized by eating in response to positive or negative emotions [1], and associated with an individual’s feelings related to eating, and perceived stress [2]. When in response to negative emotions, it has been shown to be problematic, as it often involves consuming comfort foods high in sugar or fat, which can lead to overeating or binge eating, potentially leading to weight gain or an increased body mass index (BMI) [1]. For example, among a U.S. sample of adolescents (*N* = 1622, mean age of 14.48 years, 50.6% female, and 63.8% non-Hispanic White), Kidwell et al. [3] found that EE was correlated with a frequent intake of energy-dense/nutrient-poor foods, junk food, and convenience foods, after controlling weight status. Kidwell et al. [3] also found that 30% of the study adolescents engaged in EE. Adolescents in this study with the higher rates of EE were more likely to be female and have obesity. However, Limbers et al.’s [4] systematic review found the association between increased weight and EE is either unclear or not apparent.

EE has also been linked to anxiety in adolescents [5]. According to the American Psychological Association [6], anxiety is defined by persistent and excessive worries or fears, even in the absence of a stressor. Unlike anxiety, stress is commonly caused by an external trigger. The trigger can be short-term (e.g., exam deadline) or long-term (e.g., suffering from chronic illness). Rose et al. [7] found that adolescents with higher levels of anxiety were more prone to EE compared to their lower-anxiety counterparts, as anxiety can heighten emotional sensitivity and reduce the ability to regulate impulses. The mechanisms appear to be cyclical, with the body’s stress response becoming activated because of experiencing anxiety which, in turn, increases cortisol levels. An increase in cortisol levels has been associated with the disruption of sleep patterns and an increase in high-calorie comfort food cravings [8]. Sleep disturbances, in turn, may alter hunger-regulating hormones, such as ghrelin and leptin, leading to increased appetite and preference for high-calorie foods [9,10]. The prevalence of EE varies across ethnic groups, with higher rates observed among ethnic minority adolescents compared to their non-Hispanic, White counterparts [11]. Hispanic adolescents might be at increased risk of EE due to several factors, including acculturative stress [12] and their disproportionate risk for familial financial hardship [13] or poverty [14]. Among studies of non-Hispanic children and adolescents, EE has been shown to be positively correlated with both anxiety and sleep disturbances [15].

White et al. [15] reported an association between sleep disorders and EE behaviors among teenage girls and boys, further suggesting that improved sleep may reduce the risk for EE. The direction of the relationship between anxiety, sleep, and EE in children and adolescents is unclear, though Zerón-Rugerio et al. [16] provide some evidence that adultsʹ sleep disturbances were associated with EE, and EE may mediate the relationship between inadequate sleep and obesity. Evidence also suggests that the short-term positive feelings associated with EE reinforce the behavior, making it more likely to be repeated as a coping mechanism for managing anxiety. For example, among adults, insufficient sleep impairs emotional and behavioral regulation, perpetuating the pattern of using EE to cope with anxiety [17]. Studies among adolescents appear to largely indicate no gender differences in EE [18,19]. However, girls consistently report and present higher rates of anxiety than boys, which is evident among children as young as 6 years of age [20]. There may also be gender differences in sleep patterns. For example, Fatima et al. [21] found that, among young adults, females reported poorer sleep quality than males.

A few studies have examined the relationship between EE, perceived stress, and BMI, though these have largely included adult samples. For example, Du et al. [22] examined this relationship among university students and found that EE (assessed by The Three Factor Eating Questionnaire) mediated the relationship between perceived stress and BMI for both males and females. However, an increase in sleep quality (assessed via The Pittsburgh Sleep Quality Index) weakened this association, but only among females. Likewise, in a relatively small study with 92 adults, Akkuş et al. [23] found that EE was positively associated with anxiety, depression, impulsivity, and poorer sleep quality. Furthermore, participants with obesity reported higher levels of both EE and poor sleep quality compared to those of normal weight. This study also found gender differences; women reported both higher levels of EE and poorer sleep quality than men, and poor sleep quality was directly associated with EE [23]. Finally, Rossi [24] employed a cross-sectional design involving 467 individuals from the general population (73.2% female, mean age of 31.25) and found that cognitive factors, such as thoughts related to eating behaviors, contribute to EE.

Despite prior research beginning to establish the relationship between EE and anxiety and sleep disturbances, to the authors’ knowledge, limited research has examined the relationship of anxiety, sleep quality, and EE among Hispanic children and adolescents. Focusing on Hispanic children and adolescents is important to better understand these relationships in this population and, in turn, begin to develop potential opportunities to support and intervene earlier in the development of EE. To fill this gap in literature, the present study examined the association between anxiety, sleep quality, and EE among Hispanic children and early adolescent girls and boys. Based on the reviewed literature, it was hypothesized that higher anxiety levels and poorer sleep quality would be associated with increased EE, controlling for age and BMI.

## 2. Materials and Methods

### 2.1. Participants

This study used a correctional research design. A total of 232 Hispanic children and early adolescents (*n* = 124, 53% girls; *n =* 108, 47% boys) between the ages 8 and 14 participated in this study. Girls’ mean age was 10.23 years (*SD* = 1.40), and boys’ mean age was 10.36 years (*SD* = 1.57). As shown in Table 1, most participants were born in the United States (*n* = 104, 84% girls; *n* = 94, 87% boys) and reported high acculturation levels (*n* = 88, 71% girls; *n* = 75, 69% boys). Concerning weight status, most of the participants were either overweight (*n* = 24, 19% girls; *n* = 18, 17% boys) or obese (*n* = 61, 49% girls; *n* = 61, 56% boys). The baseline data analyzed in the current study were collected between 2017 and 2019 from children and early adolescents who participated in either a summer wellness or an after-school healthy lifestyle intervention. The interventions focused on promoting nutrition education and exercise among Hispanic and African American children and early adolescents. The following inclusion criteria were employed as part of the interventions: (a) being a boy or girl of Hispanic or African American descent; (b) being between the ages of 8–14 years; (c) being at risk for being overweight or obese (due to an obesity family history) or being already overweight or obese; and (d) having no physical disability or medical condition that interfered with their engagement in nutrition and exercise components of the interventions. For this study, only the Hispanic participant data were included because the sample size of African American children and early adolescents was too small to make meaningful ethnic group comparisons. Participants in the lifestyle interventions were recruited through schoolteachers, school nurses, directors of social service agencies, medical professional referrals, and social media. The university’s Institutional Review Board approved each intervention protocol.

### 2.2. Procedure

The research team invited interested caregivers (mostly mothers, fathers, and grandmothers) and their children and early adolescents to attend a study orientation session, which was held at participants’ preferred setting, either the referent university or the participant’s school. During the study orientation, two research assistants provided caregivers and their children and early adolescents with detailed information about the study’s description, expectations, time commitment, and eligibility requirements. At this time, potential participants also had an opportunity to ask questions. At the end of the orientation, if they agreed to participate in the study, consent and assent were captured for the caregiver and their children, respectively. Subsequently, research assistants scheduled participants for two, 60-min baseline measurement sessions conducted within 2 weeks of the orientation meeting. During the baseline measurement session, after a brief introduction, research assistants provided participants with instructions and a clasp envelope, including a series of surveys regarding demographics, acculturation, emotional eating, anxiety, and sleep quality among other surveys. On average, participants completed all the surveys within 30 to 45 min and then a trained research assistant measured participants’ body height and weight individually and privately. A make-up session was scheduled within 1 week of the initial session if participants were unable either to complete surveys or did not have their height and weight measured by one of the trained research assistants.

### 2.3. Measures

#### 2.3.1. Demographic and Acculturation Surveys

The demographic survey assessed age, gender, and place of birth. Participants also completed the 12-item Short Acculturation Scale for Hispanic Youth [25], which assessed acculturation to the U.S. mainstream culture in terms of language use/proficiency (9-items, e.g., “What language do you usually speak at home?”) and social relations (3-items, e.g., “Your close friends are”) on a 5-point Likert-type scale ranging from 1 (Only Spanish) to 5 (Only English) and from 1 (All Hispanic) to 5 (All Non-Hispanic), respectively. High acculturation was determined by a sum score equal to or greater than 30 points, and low acculturation was determined by a sum score less than 30 points. In this study, the internal consistency of the acculturation measure was good (Cronbach’s α = 0.87).

#### 2.3.2. Weight Status

Trained research assistants measured each participant’s body height using the Seca 213 stadiometer and rounded to the nearest 0.1 cm. Body weight was measured using a scale (Tanita TBF 310) and rounded to the nearest 0.1 kg. Participants’ body weight and height were taken while wearing light clothes without shoes and hair without ponytail. Participants’ objectively measured weight and height were used to determine their body mass index (BMI), which is calculated by weight (in kilograms) divided by the square of height (in meters). In addition, we plotted the participants’ weight and height on the Centers for Disease Control and Prevention growth charts for age- and gender-specific percentiles to determine the BMI percentile [26]. Based on these BMI percentile growth charts, participants were classified as underweight with a calculated BMI of <5th percentile; normal or healthy weight with a BMI between the 5th and 84th percentile; overweight with a BMI between the 85th–94th percentile; and with obesity with a BMI of ≥95th percentile.

#### 2.3.3. Emotional Eating

EE was assessed using the McKnight Risk Factor Survey-IV [27] EE subscale. Following McKnight investigators’ scoring guidelines, the EE score was computed by averaging responses to the six EE items. Three out of the six items assessed eating less (e.g., "In the past month, how often did you eat less than usual when you were bored or to try to feel better about self, and when you were upset?”). The other three items assessed eating more (e.g., “In the past month, how often did you eat more than usual when you were bored, trying to feel better about self, and when you were upset?”). Items from this subscale were rated using a 5-point Likert-type scale (1 = never to 5 = always). In this study, the internal consistency of the EE subscale was good (Cronbach’s α = 0.88).

#### 2.3.4. Multidimensional Anxiety Scale for Children 2nd Edition (MASC 2^TM^)

MASC 2 ^TM^ is a comprehensive measure of anxiety-related symptoms in children and adolescents aged 8 to 19 years [28]. This measure consists of 50 items that assess a broad range of emotional, physical, cognitive, and behavioral symptoms of anxiety. Participants answered items on a 4-point Likert-type scale, ranging from 0 (never true about me) to 3 (often true about me). The questionnaire yields both raw scores and standardized *T*-scores to assess the overall degree of self-reported anxiety. In the present study, *T*-scores were used to evaluate participants’ levels of anxiety. The MASC 2^TM is^ a widely recognized and psychometrically sound measure of anxiety, with excellent internal consistency (Cronbach’s α = 0.92) in its normative sample of 8–19-year-olds [29]. In the present study, the internal consistency of MASC 2 was similarly strong (Cronbach’s α = 0.94).

#### 2.3.5. Pittsburgh Sleep Quality Index (PSQI)

The PSQI is a 19-item self-report questionnaire, which yields a global PSQI score indicating sleep quality [30]. The current study used the global PSQI score to determine sleep quality. Global PSQI scores range from 0 to 21, with lower scores than 5 indicating a healthy sleep quality; whereas scores equal to or greater than 6 are indicative of poor sleep quality. Raniti et al. [31] reported that the internal consistency of the global PSQI among adolescents was acceptable (Cronbach’s α = 0.73). In the present study, the internal consistency of the global PSQI was good (Cronbach’s α = 0.83).

### 2.4. Statistical Analysis

Descriptive characteristics of study participants were calculated using means and standard deviations for continuous variables and percentages for categorical variables. Independent sample *t*-tests were conducted to examine gender differences in BMI, anxiety, sleep quality, and EE. Pearson correlations were computed separately for boys and girls to examine relationships among EE, anxiety, sleep quality, age, and BMI. The *t*-tests and correlations guided the final regression model. To test our hypothesis, two hierarchical multiple regressions were conducted separately for Hispanic girls and boys to test to what extent anxiety and sleep quality were associated with EE. In Model 1, age and BMI were entered as control variables. In Model 2, anxiety was added to examine its unique contribution to the outcome. In Model 3, sleep quality was added to determine its additional contribution beyond anxiety. The hierarchical approach allowed for examination of the unique variance explained by each predictor variable, while controlling for previously entered variables. For all analyses, standardized beta coefficients (*β*) were reported along with their associated *p*-values. To evaluate whether the regression lines varied by gender, we conducted an additional univariate analysis of covariance (ANCOVA), using gender as the between-subjects factor and age, BMI, anxiety, and sleep quality as covariates. Analyses were conducted using SPSS Statistics version 29.0 with a statistical significance set at *p* < 0.05. In addition to *p*-values, we computed Cohen’s *d* for all between-group mean comparisons to quantify the standardized mean difference. By convention, *d* = 0.20 is a small effect, *d* = 0.50 a medium effect, and *d* = 0.80 is a large effect. Prior to fitting the hierarchical regressions, we evaluated multicollinearity by examining tolerance and variance inflation factors (VIF) for all predictors, following Tabachnick and Fidell [32]. In each gender-stratified model, tolerance values exceeded 0.82 (range 0.82–0.95) and VIFs were below 1.25 (range 1.05–1.22), indicating no problematic collinearity. Residuals were inspected via histogram and scatterplots of standardized residuals vs. predicted values, confirming approximate normality and homoscedasticity.

## 3. Results

### 3.1. Gender Differences Across Key Variables

Results of the independent samples *t*-tests indicated that there were no significant gender differences in BMI between boys (*M* = 24.26, *SD* = 5.94) and girls (*M* = 24.50, *SD* = 7.13), *t* (226) = −0.273, *p* = 0.785, Cohen’s *d* = −0.04. Similarly, anxiety levels showed no significant differences between boys (*M* = 51.81, *SD* = 12.28) and girls (*M* = 50.92, *SD* = 13.00), *t* (204) = 0.501, *p* = 0.617, Cohen’s *d* = 0.07. No significant gender differences were found in sleep quality between boys (*M* = 3.80, *SD* = 3.58) and girls (*M* = 3.24, *SD* = 2.90), *t* (229) = 1.313, *p* = 0.190, Cohen’s *d* = 0.17. However, a significant gender difference was found in EE, with boys reporting significantly higher levels (*M* = 1.84, *SD* = 0.94) compared to girls (*M* = 1.62, *SD* = 0.65), *t* (206) = 1.994, *p* = 0.047, Cohen’s *d* = 0.28, which can be interpreted as a small effect [33]. That is, boys reported higher EE levels compared to girls, but there were no significant gender differences in sleep quality and anxiety scores. Nevertheless, because this is an understudied area, and prior research suggests that it may be important to explore the relationships separately for girls and boys [3], subsequent analyses were conducted separately by gender.

### 3.2. Correlations Across Key Variables

Pearson correlations were examined to determine which predictor variables should be included in the hierarchical regression models. Significant correlations were detected for EE, anxiety, sleep quality, age, and BMI for both boys and girls. For girls, anxiety was positively correlated with EE (*r* = 0.282, *p* < 0.01) and with sleep quality (*r* = 0.497, *p* < 0.001) but not with age (*r* = −0.032, *p* = 0.740) or BMI (r = −0.103, *p* = 0.284). Likewise, sleep quality was correlated with EE (*r* = 0.403, *p* < 0.001) but not with age (*r* = 0.149, *p* = 0.099) or BMI (*r* = 0.103, *p* = 0.260). Age was only associated with BMI (*r* = 0.417, *p* < 0.001), with older girls tending to have a higher BMI. For boys, a similar pattern was observed; anxiety correlated with EE (*r* = 0.391, *p* < 0.001) and with sleep quality (*r* = 0.431, *p* < 0.001) but not with age (*r* = 0.010, *p* = 0.928) or BMI (*r* = 0.142, *p* = 0.176). Sleep quality in boys was correlated with EE (*r* = 0.416, *p* < 0.001) and BMI (*r* = 0.307, *p* < 0.001) but not with age (*r* = 0.038, *p* = 0.696). Like girls, age was significantly correlated with BMI (*r* = 0.365, *p* < 0.001), with older boys tending to have a higher BMI.

### 3.3. Factors Associated with Emotional Eating in Hispanic Girls and Boys

Based on significant correlations to assess the extent to which anxiety and sleep quality were associated with EE in Hispanic girls and boys (controlling for age and BMI), two hierarchical regressions were conducted—one for girls and one for boys (Table 2). Due to missing data across key variables, we opted for listwise deletion for the regression analyses, reducing the sample to *n* = 111 for girls and *n* = 92 for boys. Specifically, for girls, missing data were 0% for age, 0.8% for BMI and sleep quality, 10% for anxiety, and 10% for EE. For boys, the missing data were 0% for age, 3% for BMI, 13% for anxiety, 0% for sleep quality, and 14% for EE. For girls, Model 1 included age and BMI as factors associated with EE and was not statistically different from zero [*R*^2^ = 0.011 (adjusted *R*^2^ = −0.007, F(2, 108) = 0.606, *p* = 0.547]. In Model 2, anxiety was added and was a significant factor associated with EE (*β* = 0.301, *p* < 0.01), controlling age and BMI. This predictor model explained 7.5% of the variance in EE [*R^2^* = 0.101, adjusted *R*^2^ = 0.075, *F*(3, 107) = 3.99, *p =* 0.010]. More specifically, this indicates that 7.5% of the variance in EE scores among girls was predicted by BMI, age, and anxiety. In Model 3, sleep quality was added to the model, which significantly improved the model. The addition of sleep quality resulted in the full model explaining 15.9% of the variance in EE [*R*^2^ = 0.189, adjusted *R*^2^ = 0.159, *F*(4, 106) = 6.194, *p* < 0.001], resulting in a significant incremental change in *R*^2^. Sleep quality was a significant predictor of EE (*β* = 0.350, *p* < 0.001); whereas the effect of anxiety became nonsignificant (*β* = 0.122, *p* = 0.234).

For boys, Model 1 also included age and BMI, and like the girl-only sample, this model was not statistically different from zero [*R*^2^ = 0.053, adjusted *R*^2^ = 0.031, *F*(2, 89) = 2.473, *p* = 0.090]. In Model 2, anxiety was added and was a significant predictor (*β* = 0.358, *p*< 0.001), with BMI, age, and anxiety explaining 15.0% of the total variance in EE scores [*R*^2^ = 0.178, adjusted *R*^2^ = 0.150, *F*(3, 88) = 6.355, *p* < 0.001]. In Model 3, sleep quality was added, which resulted in the full model explaining 20.6% of the variance in EE [*R*^2^ = 0.241, adjusted *R*^2^ = 0.206, *F*(4, 87) = 6.910, *p* < 0.001]. Sleep quality (*β* = 0.302, *p* < 0.01) and anxiety (*β* = 0.247, *p* < 0.05) were both significant independent predictors of the variance in EE scores among boys, controlling BMI and age. Unlike in girls, anxiety remained a significant predictor for boys, suggesting that both anxiety and sleep quality independently predicted EE in boys.

To examine whether the regression lines differed by gender, we ran an ANCOVA with gender as the between-subjects factor and age, BMI, anxiety, and sleep quality as covariates. The overall model was significant, *F*(5, 197) = 11.41, *p* < 0.001. However, significant gender differences were not detected.

## 4. Discussion

This study examined the association of anxiety and sleep quality to EE in a sample of Hispanic girls and boys. The researchers hypothesized that high anxiety scores and poor sleeping quality would contribute to EE after controlling for age and BMI. Results partially supported this hypothesis, revealing that anxiety scores and sleep quality were associated with EE. In the regression analyses, sleep quality emerged as the sole significant factor related to EE for girls; whereas, for boys, both anxiety and sleep quality were significantly and independently associated with EE. While anxiety has been linked to EE in children and adolescents overall [18,34], the current study found this positive association was significant only for Hispanic boys and not Hispanic girls. This finding was surprising and may reflect distinct gender socialization patterns and coping strategies. For instance, Hispanic girls may have different socialization experiences that shape how they express and cope with anxiety. Girls may share their anxiety with family and friends [35]. In contrast, Hispanic boys may be less likely to express anxiety openly [36], potentially leading to greater reliance on EE as a maladaptive coping strategy.

Consistent with previous research [37,38], our results also revealed that sleep quality was a significant factor associated with EE scores for Hispanic girls and boys. Poor sleep quality may disrupt emotional regulation (e.g., decreased self-control, increased impulsivity), potentially leading to increased vulnerability to using food as a coping mechanism [39]. Addressing sleep-related self-regulation deficits could be a crucial component in interventions aimed at promoting both emotional well-being and healthier lifestyle choices.

The findings of this study should be interpreted considering several limitations. First, the generalizability of the results is limited, because the sample consisted of primarily Hispanic children and early adolescents with overweight or obesity, who were born in the United States, highly acculturated, and sought participation in a healthy lifestyle intervention. Future studies should examine the independent and additive effects of Hispanic children and early adolescents’ characteristics, including samples of Hispanic girls and boys with varying levels of weight status and acculturation. Second, the cross-sectional nature of this study impeded the assessment of bidirectional or causal relationships. Future studies that utilize a longitudinal design are warranted to elucidate the temporal direction of the relationship between Hispanic children and early adolescents’ anxiety, sleep quality, and EE. For example, longitudinal designs that follow children and adolescents over time can help clarify the direction of associations and show how EE patterns may change as children and adolescents get older. In addition, using objective measures, such as sleep tracking devices and lab-based eating assessments, could give a fuller picture of this relationship.

Third, a small amount of variance was accounted for in these analyses, which suggests that other variables (e.g., dietary restrictions, parental control, and parental appearance teasing) may be associated with EE among Hispanic girls and boys. For instance, a study examining EE patterns in Mexican adolescents between 15 and 17 years old found that dietary restrictions, weight concerns, and higher parental control over eating were significantly associated with EE behaviors [40]. In another study, Webb et al. [41] found that social anxiety symptoms and parental appearance teasing were also associated with EE in both adolescent girls and boys. Rossi [24] reported that EE was highlighted as associated with food addiction. Future studies should assess how food addiction and familial pressures influence dietary restrictions, body image, and teasing in Hispanic children and preadolescents’ EE. Understanding additional predictors of EE may enable more precise tailoring of identification and interventions to address and support those Hispanic children and adolescents with EE. Fourth, we acknowledge the limitations of using dated data, particularly analyzing data collected prior to the COVID-19 pandemic. Specifically, such data may not reflect current trends or patterns of behaviors that may have been impacted by the COVID-19 pandemic. For example, data collected prior to the COVID-19 pandemic might be missing newly emerging variables or factors which have the potential to limit analysis or prediction accuracy. Nevertheless, because limited information is available regarding Hispanic children and preadolescents’ EE and its correlates, the findings of this study contribute to the body of knowledge regarding the factors associated with EE in this population.

These limitations are offset by several study strengths. The sample consisted entirely of Hispanic children and early adolescents. This continues to be an understudied population that appears to be at an increased risk for EE [11,12]. Given the predictions that the number of Hispanic-identifying individuals in the U.S. will continue to increase [42] and unique cultural aspects, it is especially important to continue to include those who identify as Hispanic to better tailor prevention and intervention programs. Furthermore, the study of anxiety and sleep quality in relation to EE has practical implications. More specifically, the early identification of EE should consider sleep quality in Hispanic children and early adolescent girls and boys as a screening variable to better identify the need for tailored interventions. Pediatricians, school health providers, and mental health professionals should routinely assess sleep quality and provide education and resources to improve sleep hygiene among Hispanic children and early adolescents. Further, anxiety screening and management may be especially important for Hispanic boys in the context of EE.

Additionally, based on the current study and previous work, providers should be attuned to cultural factors that may influence the expression of anxiety and consider culturally sensitive approaches to assessment and interventions to reduce EE. For boys, strategies to cope with anxiety and stress may be key. For girls, a focus on sleep may be more impactful. Based on the analyses, both girls and boys may benefit from learning emotion regulation skills and healthy coping mechanisms. Although speculative, a family-based approach may be particularly effective for Hispanic children and early adolescents, given the central role of family and food in Hispanic culture [43]. For example, engaging caregivers in promoting healthy sleeping habits, modeling adaptive coping, and fostering positive family food dynamics could help reduce the incidence of EE among Hispanic children and early adolescents. However, an investigation of cultural influence related to EE among Hispanic children and adolescents is needed.

Additionally, food plays a major role in family life, with cultural practices, traditions and meanings shaping attitudes and behaviors around eating from an early age [44]. For example, large family meals and favorite dishes often become associated with bonding, celebrating, and expressions of love. Over time, these deep-rooted cultural influences become intertwined with biological and psychological factors that can trigger EE [45]. The link between culturally meaningful foods and soothing feelings may lead individuals to eat to manage emotions rather than to satisfy physical hunger. This may be further exacerbated by the link between experiencing daily discrimination and subsequent sleep disturbances [46].

Schools serving Hispanic children and adolescents should consider incorporating education on sleep, emotional health, nutrition, and coping skills of health curricula and prevention programs. For instance, a 12-week school mindfulness-based diet and exercise intervention reported a reduction in EE, anxiety, and sleep latency post-intervention among Latinx and Black preadolescent girls and boys [47]. Thus, additional consideration and exploration of what interventions, programs, and support may be developed should include roles for caregivers or other family members to support children and adolescents.

## 5. Conclusions

The current study is a necessary first step in examining factors associated with EE among Hispanic children and early adolescents. The findings suggest that sleep quality has a significant and positive association with EE for both girls and boys. However, anxiety was associated with EE in Hispanic boys only. These findings suggest that investigators and health professionals particularly focused on developing interventions aimed at improving eating behaviors and managing weight in Hispanic children and early adolescents should tailor these to address anxiety and sleep quality and their association with EE.

## Figures and Tables

**Table 1:** Sample descriptive characteristics by gender.

Characteristics	Girls (*n* = 124)	Boys (*n* = 108)
Age (years)		
8–9	40 (32%)	42 (39%)
10–12	75 (61%)	53 (49%)
13–14	9 (7%)	13 (12%)
Missing	0	0
Place of Birth		
USA	104 (84%)	94 (87%)
Mexico	5 (4%)	4 (4%)
Central America	2 (2%)	2 (2%)
Other	4 (3%)	0
Missing	9(7%)	8 (7%)
Acculturation		
Low	20 (16%)	18 (17%)
High	88 (71%)	75 (69%)
Missing	16 (13%)	15 (14%)
Weight Status		
Underweight	3 (2%)	3 (3%)
Healthy weight	35 (28%)	22 (20%)
Overweight	24 (19%)	18 (17%)
Obese	61 (49%)	61 (56%)
Missing	1 (2%)	4 (4%)

Table footer: Participants whose place of birth is categorized as ‘Other’ refers to individuals from South America; percentages were round up. Data are presented as *n* (%).

**Table 2:** Hierarchical regression analysis predicting emotional eating in Hispanic children and early adolescents.

Variable	Girls (*n* = 111)					Boys (*n* = 92)				
	B	β	t	*R* ^2^	Adj.R^2^	B	β	t	*R* ^2^	Adj.R^2^
Model 1				0.011	−0.007				0.053	0.031
Age	−0.026	−0.057	−0.547			−0.133	−0.222	−1.928		
BMI	0.010	0.115	1.095			0.033	0.212	1.845		
Model 2				0.101 ***	0.075 ***				0.178 ***	0.150 ***
Age	−0.027	−0.061	−0.609			−0.120	−0.198	−1.840		
BMI	0.013	0.147	1.459			0.024	0.151	1.384		
Anxiety	0.015	0.301 **	3.262			0.027	0.358 ***	3.665		
Model 3				0.189 ***	0.159 ***				0.241 ***	0.206 ***
Age	−0.047	−0.105	−1.083			−0.090	−0.149	−1.408		
BMI	0.011	0.125	1.293			0.005	0.029	0.254		
Anxiety	0.006	0.122	1.197			0.019	0.247 *	2.393		
Sleep Q	0.079	0.350 ***	3.410			0.080	0.302 **	2.688		

Table footer: * *p* < 0.05, ** *p* < 0.01, *** *p* < 0.001 BMI = body mass index, Sleep Q = sleep quality.

## Data Availability

The data are not publicly available due to concerns regarding privacy.
